# Complicated biliary disease during pregnancy: a retrospective cohort analysis from a tertiary care facility

**DOI:** 10.1007/s00464-025-12329-5

**Published:** 2025-11-03

**Authors:** Amrita Iyer, Sunjay Kumar, Isabella Muti, Edoardo Manca, Sami Tannouri, Talar Tatarian, Vincenzo Berghella, Francesco Palazzo

**Affiliations:** 1 Department of Surgery, Thomas Jefferson University Hospital, 1100 Walnut Street 5th Floor, Philadelphia, PA 19107 USA; 2 Department of Obstetrics and Gynecology, Thomas Jefferson University Hospital, Philadelphia, PA 19107 USA

**Keywords:** Complicated biliary disease, Pregnancy, Cholecystectomy, Surgical outcomes, Adverse birth outcomes, Healthcare utilization

## Abstract

**Background:**

Symptomatic biliary disease frequently prompts patients to seek medical care during pregnancy. Most guidelines now recommend prompt surgical intervention regardless of trimester for complicated biliary disease during pregnancy (i.e., acute cholecystitis, choledocholithiasis, cholangitis, and gallstone pancreatitis). We investigated our health system’s experience with the care of this patient population in the context of current recommendations.

**Methods:**

The electronic medical record of a large, multi-hospital health system was queried from January 2017 to December 2024 for all patients with diagnoses of pregnancy and complicated biliary disease. Patients were divided into 4 groups based on trimester at time of procedural intervention or postpartum management. Patients in the postpartum group presented with symptomatic biliary disease during their pregnancy and progressed to complicated disease within three months after delivery, necessitating procedural intervention in that period of time.

**Results:**

The search yielded 48 patients: 7 first trimester, 22 s, 5 third, and 14 postpartum. Procedural interventions included ERCPs and laparoscopic cholecystectomies. All cholecystectomies were completed without major surgical morbidity. Most interventions occurred in the second trimester or postpartum (75%). Surgical outcomes were similar across all groups; any variability in proportions was attributable to low sample sizes. Parental-fetal outcomes, including incidence of preeclampsia, pregnancy loss, and preterm delivery, were similar across groups.

**Conclusion:**

Our analysis confirms that procedural interventions for complicated biliary disease are safe to perform regardless of trimester. Our work also demonstrates the difficulties of studying this disease process as the prevalence is relatively low. This area of study could benefit from multi-institutional collaboration.

**Graphical abstract:**

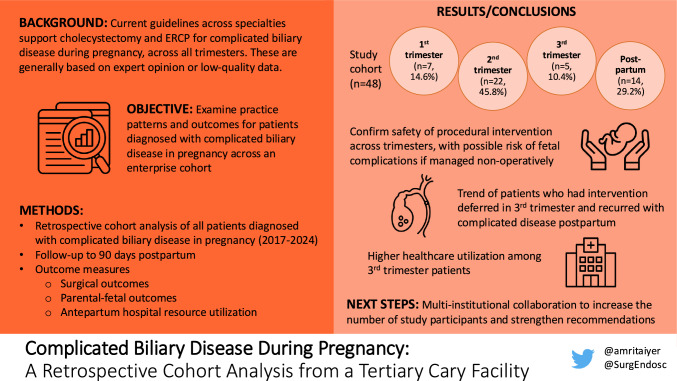

Symptomatic biliary disease is a common indication for non-obstetric procedures during pregnancy, affecting 30,000 patients in the US annually [1–5]. Complicated biliary disease in particular is worrisome in pregnancy given its association with serious surgical and parental-fetal complications, including maternal mortality [3, 6, 7]. Operative management of complicated biliary disease may mitigate risks of parental-fetal complications when compared to non-operative management [3, 8, 9].

While surgery was traditionally avoided in the 1st and 3rd trimesters due to concerns for associated adverse obstetric outcomes, growing evidence from the past two decades has indicated that cholecystectomy is safe to perform in all trimesters [3, 4, 10, 11]. Consequently, updated clinical guidelines across multiple specialties now recommend prompt procedural intervention regardless of trimester [12–14]. For example, the Society of American Gastrointestinal and Endoscopic Surgeons (SAGES) has issued successive guidelines in 2011, 2017, and again in 2024 stating that “laparoscopic cholecystectomy is the treatment of choice in the pregnant patient with symptomatic gallbladder disease, regardless of trimester (quality of evidence: low; strength of recommendation: weak)” [12, 15, 16].

Notably, the aforementioned guidelines for management of biliary disease in pregnancy fall short of high-strength recommendations, at times citing that “evidence [remains] low quality with an overall low certainty” [12]. The 2024 SAGES guidelines, for example, relied in part on expert panel decision-making due to a considerable lack of robust, high-quality studies [12]. Large database analyses are often missing details such as the trimester of pregnancy, while single institution retrospective reviews frequently pool outcomes across cases of both uncomplicated and complicated biliary disease [8, 11, 17–21]. Additionally, many of these retrospective studies group patients by intervention before delivery, providing little insight into the natural history of gallstone disease extending into the postpartum period and leaving key questions about optimal timing of care unanswered.

Pregnant patients with complicated biliary disease remain a vulnerable and therefore challenging group to study. Furthermore, the currently limited and inconsistent evidence base poses a substantial barrier to developing high-strength recommendations for managing this disease process. Collecting clinically robust data on surgical and parental-fetal outcomes is necessary to help strengthen treatment guidelines. Additional insight is also needed regarding the impact of the timing of intervention on levels of healthcare utilization, and subsequently, overall financial burden. In this study, we aim to address these gaps by investigating our health system’s experience with pregnant patients diagnosed with complicated biliary disease and the associated clinical outcomes.

## Methods

We conducted a retrospective review of all pregnant patients treated for biliary disease at Thomas Jefferson University Hospital (a large academic medical center) and seven affiliate centers (including three smaller academic and four community hospitals) between January 2017 and December 2024. Inclusion criteria included adult patients (≥ 18 years old) who presented to the emergency department (ED) or outpatient clinic with symptomatic biliary disease while pregnant. Patients who were diagnosed on index admission with complicated biliary disease (defined as acute cholecystitis, cholangitis, choledocholithiasis, or gallstone pancreatitis), and patients who progressed to complicated biliary disease over the course of their pregnancy, were included in our analysis. Patients who were diagnosed with only symptomatic, uncomplicated biliary disease (i.e., symptoms of biliary colic with evidence of cholelithiasis and/or sludge on ultrasound) were excluded from the study.

Patients were divided into four groups based on trimester at the time of procedural intervention (for those patients who underwent intervention antepartum) or postpartum management. Patients in the postpartum group were those who had presented with symptomatic biliary disease during their pregnancy and progressed to complicated disease within 90 days after delivery, necessitating procedural intervention in that time, or developed complicated disease leading to induction and subsequent cholecystectomy. Biliary diseases were defined by the final diagnosis at time of discharge. Specifically, acute cholecystitis was carefully defined by the presence of fever, right upper quadrant or epigastric pain, and leukocytosis. If any of these criteria were lacking, confirmatory imaging findings or final pathology consistent with acute cholecystitis were necessary to confirm the diagnosis. Acute cholangitis was defined using diagnostic criteria from the 2018 Tokyo Guidelines [22]. Choledocholithiasis was defined as presence of gallstones in the common bile duct as proven by magnetic resonance cholangiopancreatography or endoscopic retrograde cholangiopancreatography (ERCP). Gallstone pancreatitis was defined as the presence of gallstones and/or biliary ductal dilation on imaging in addition to acute pancreatitis as set forth by criteria delineated in the 2012 Revised Atlanta Classification [23].

Antepartum biliary history was documented for each patient, including number of prior episodes, ED visits, admissions, and total length of stay. The number of prior episodes was defined as distinct healthcare encounters, including any visits to an outpatient clinic or the ED for symptoms related to the patient’s biliary colic. ED visits included visits to the emergency department as well as admission to observation with hospital stay < two midnights and visits to obstetric triage. Admissions were defined as any hospital stay ≥ two midnights. Antepartum length of stay was calculated as the total number of midnights patients spent in the hospital for biliary disease while pregnant.

Surgical outcomes included post-surgical length of stay, common bile duct injury, cystic duct leak, post-operative ERCP, post-operative interventional radiology drain, 30-day follow-up ED visits, and 30-day follow-up readmissions. Parental outcomes included intrapartum or postpartum hemorrhage, intrahepatic cholestasis of pregnancy, preeclampsia, eclampsia, pregnancy loss, preterm delivery, and Cesarean sections for fetal distress. Fetal outcomes included birthweight and weight for gestational age, Apgar scores at 1 and 5 min, fetal growth restriction, and NICU admissions. To capture relevant time intervals, the following dates were collected for every patient: date of first episode of biliary colic, date of procedural intervention, and date of delivery. For those patients who underwent both an ERCP and a cholecystectomy antepartum, time from intervention to delivery was calculated using the date of cholecystectomy.

Descriptive statistics were used to compare the four groups, after excluding missing data. The study protocol was ruled exempt by the Thomas Jefferson University Institutional Review Board.

## Results

**Fig. 1:**
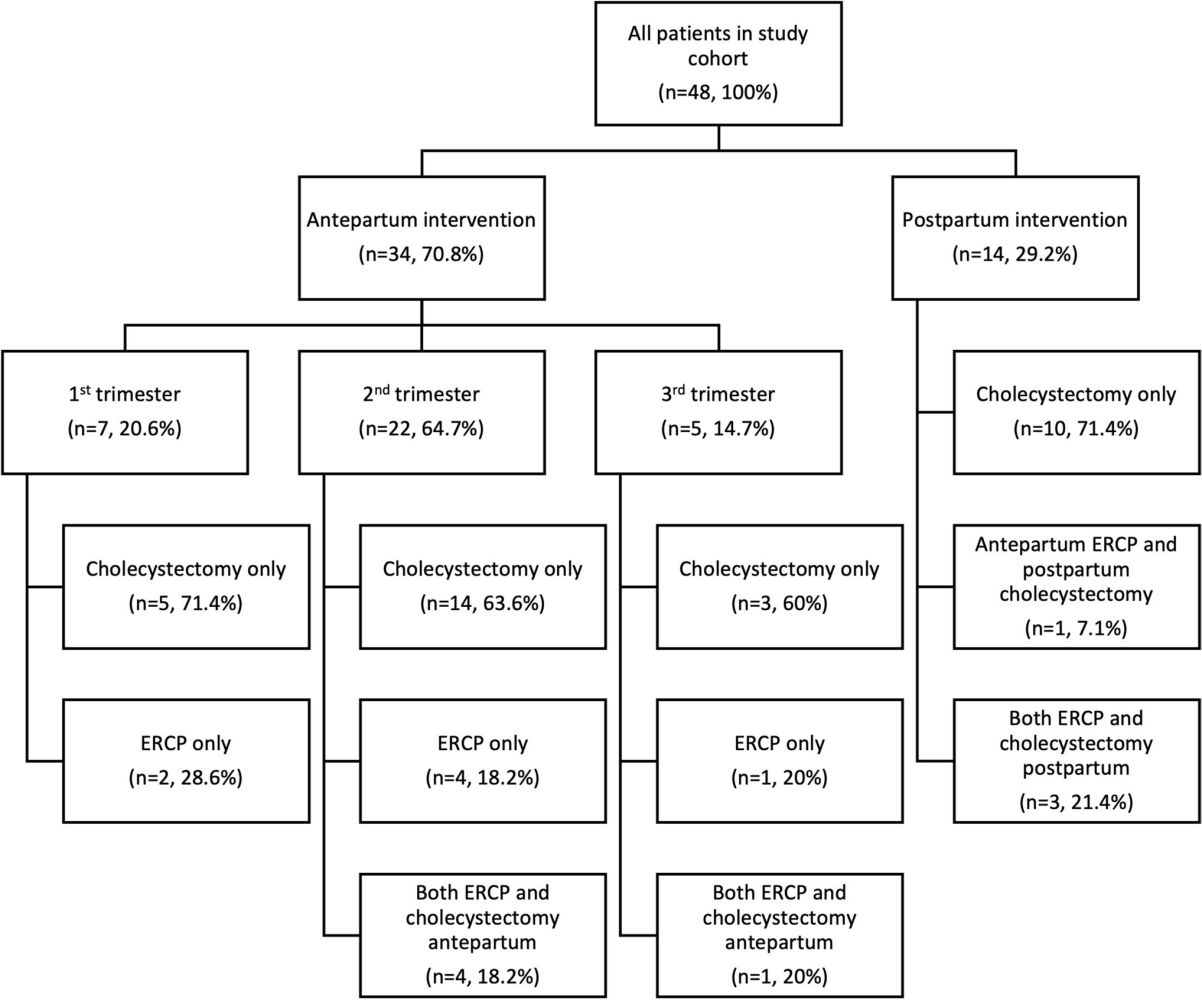
Management of patients diagnosed with complicated biliary disease during pregnancy. *ERCP* endoscopic retrograde cholangiopancreatography

Our search identified 48 pregnant patients diagnosed with complicated biliary disease during the study period, as shown in Fig. 1. 34 patients (70.8%) underwent a procedural intervention during their pregnancy. 7 (20.6%) patients underwent an intervention in their 1st trimester, 22 (64.7%) in their 2nd trimester, and 5 (14.7%) in their 3rd trimester. 14 patients (29.2%) were managed non-operatively during their pregnancy and underwent a postpartum intervention. Baseline characteristics such as age at delivery, pre-pregnancy BMI, medical history, and comorbidities were similar between the two cohorts (Table 1). Biliary diagnoses included acute cholecystitis (*n* = 18), choledocholithiasis (*n* = 12), cholangitis (*n* = 1), and gallstone pancreatitis (*n* = 17); a breakdown of diagnoses by timing of procedural intervention can be found in Table 2. Procedural interventions included a combination of ERCPs and/or cholecystectomy, done in the antepartum and/or postpartum periods (Fig. 1). All cholecystectomies were performed minimally invasively; no cases were converted to open.

**Table 1:** Demographic characteristics and medical comorbidities by timing of intervention

	Antepartum (*n* = 34)			Postpartum (*n* = 14)
	1st trimester (*n* = 7)	2nd trimester (*n* = 22)	3rd trimester (*n* = 5)	
Age at delivery, median (range)	31 (22 to 42)			29 (19 to 35)
	31 (26 to 39)	31 (22 to 42)	31 (23 to 37)	
Pre-pregnancy BMI, median (range)	32.60 (20.16 to 46.94)			32.89 (21.28 to 46.36)
	29.50 (24.89 to 37.79)	33.10 (20.16 to 46.94)	32.61 (28.35 to 32.81)	
Smoking history: active smoker	1 (14.3%)	1 (4.8%)	0	1 (7.1%)
Smoking history: former smoker	2 (28.6%)	4 (19%)	1 (20%)	1 (7.1%)
Medical history and comorbidities				
Diabetes mellitus	0	1 (4.8%)	0	1 (7.1%)
Pancreatitis	0	1 (4.8%)	0	1 (7.1%)
Peptic ulcer disease	0	1 (4.8%)	0	1 (7.1%)
GERD	1 (14.3%)	4 (19%)	0	1 (7.1%)
Substance use	0	1 (4.8%)	0	0
Preeclampsia	0	3 (14.3%)	1 (20%)	0
Cesarean section	0	4 (19%)	3 (60%)	2 (14.3%)

*BMI* body mass index, *GERD* gastroesophageal reflux disease

**Table 2:** Complicated biliary disease diagnoses at time of discharge, by timing of intervention

	1st trimester (*n* = 7)	2nd trimester (*n* = 22)	3rd trimester (*n* = 5)	Postpartum (*n* = 14)
Acute cholecystitis	5 (71.4%)	8 (36.4%)	1 (20%)	4 (28.6%)
Choledocholithiasis	2 (28.6%)	6 (27.3%)	2 (40%)	2 (14.3%)
Cholangitis	0	1 (4.5%)	0	0
Gallstone pancreatitis	0	6 (31.8%)	2 (40%)	8 (57.1%)

**Fig. 2:**
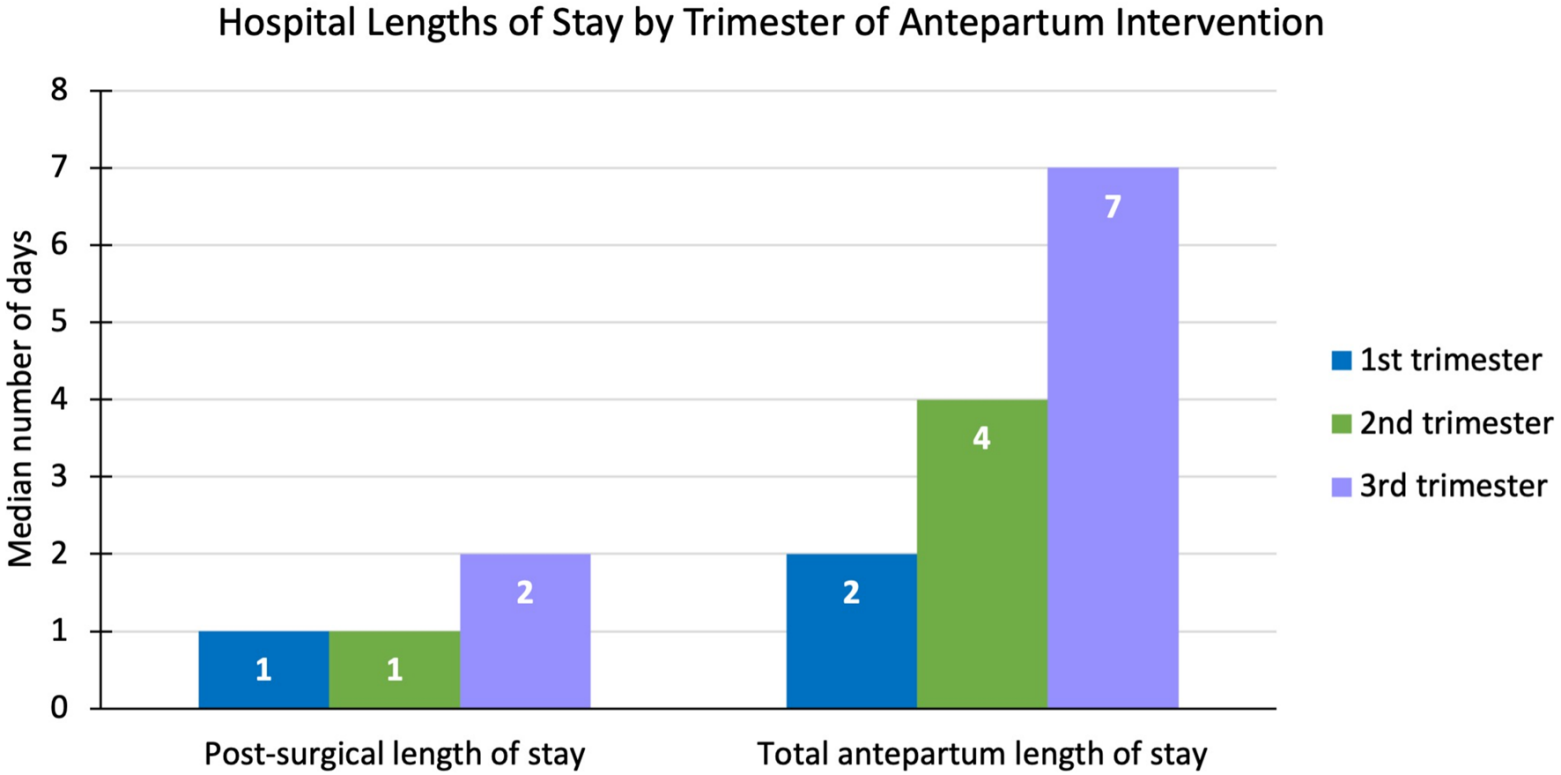
Healthcare utilization by trimester of antepartum intervention

The median number of prior episodes during pregnancy were similar in the antepartum (1 episode) and postpartum (2 episodes) intervention cohorts (Table 3). Both the antepartum and postpartum cohorts had a median of one ED visit. Median total antepartum length of stay was understandably higher for those patients undergoing antepartum intervention (4 midnights), compared to those undergoing postpartum intervention (1 midnight). Total antepartum lengths of stay increased for each subsequent trimester (Fig. 2).

**Table 3:** Antepartum healthcare encounters for symptomatic biliary disease, by timing of intervention

	Antepartum (*n* = 34)			Postpartum (*n* = 14)
	1st trimester (*n* = 7)	2nd trimester (*n* = 22)	3rd trimester (*n* = 5)	
Number of prior episodes, median (range)	1 (1 to 4)			2 (1 to 4)
	1 (1 to 2)	1 (1 to 3)	2 (1 to 4)	
Number of ED visits, median (range)	1 (1 to 4)			1 (0 to 4)
	1 (1 to 2)	1 (1 to 3)	2 (1 to 4)	
Number of admissions, median (range)	1 (0 to 3)			1 (0 to 2)
	1 (1 to 1)	1 (0 to 2)	1 (1 to 3)	
Total length of stay, median (range)	4 (0 to 3)			1 (0 to 7)
	2 (1 to 5)	4 (0 to 13)	7 (4 to 13)

Patients in both antepartum and postpartum intervention cohorts had a median post-surgical length of stay of one midnight, with higher median stay for patients in the 3rd trimester compared to 1st and 2nd trimester patients (Table 4). One patient (7.1%) in the postpartum intervention cohort had a cystic duct leak as identified on a HIDA scan after presentation to the ED four days post-operatively. The patient immediately underwent an ERCP with sphincterotomy and biliary and pancreatic stent placement but did not require a hospital admission. There were no common bile duct injuries, and no patients required a percutaneous drain placed post-operatively. As expected, median number of days from intervention to delivery were highest in the 1st trimester subgroup (202 days vs. 149 days 2nd trimester subgroup vs. 41 days 3rd trimester). The median time from delivery to postpartum intervention was 27 days. Other post-operative outcomes are presented in Table 4, with no major differences between groups.

**Table 4:** Surgical outcomes by timing of intervention

	Antepartum (*n* = 34)			Postpartum (*n* = 14)
	1st trimester (*n* = 7)	2nd trimester (*n* = 22)	3rd trimester (*n* = 5)	
Post-surgical length of stay, median (range)	1 (0 to 5)			1 (0 to 3)
	1 (0 to 2)	1 (0 to 3)	2 (1 to 5)	
Cystic duct leak	0	0	0	1 (7.1%)
Post-op ERCP	0	0	0	1 (7.1%)
30-day ED visits	2 (28.6%)	2 (10%)	2 (50%)	1 (7.1%)
Reasons for 30-day ED visits	1. Nonspecific chest pain (VTE/ACS ruled out) 2. Missed abortion (GA 7w4d, 32 days post-operatively	Nonspecific abdominal pain (both patients)	1 Eclamptic seizure 2. Labor (GA 34w, 6 days post-op)	1. Abdominal pain (cystic duct leak)
30-day readmissions	0	2 (10%)	2 (50%)	0
Reasons for 30-day readmissions	N/A	Nonspecific abdominal pain, no final definite diagnosis (both patients)	1. Management of eclampsia 2. Operative vaginal delivery	N/A

*GA* gestational age

Parental-fetal outcomes can be found in Table 5. There were no cases of parental mortality. Four patients (28.6%) were induced for severity of symptoms associated with their biliary disease, and subsequently underwent a cholecystectomy. One 1st trimester patient (14.3%) experienced a pregnancy loss. One 2nd trimester patient (5.6%), two 3rd trimester patients (50%), and three postpartum patients (21.4%) had preterm deliveries. Three 2nd trimester patients (17.6%) and two postpartum patients (15.4%) underwent C-sections for fetal distress. Fetal outcomes can also be found in Table 5. There were no neonatal deaths. Rates of NICU admissions are as follows: 12.5% 2nd trimester (*n* = 2), 25% 3rd trimester (*n* = 1), 41.7% postpartum (*n* = 5). Median Apgar scores at 1 and 5 min were similar between the antepartum and postpartum cohorts and across trimesters. Median birthweights were lower with each subsequent trimester (3490g 1st trimester vs. 3330g 2nd trimester vs. 2736g 3rd trimester). 7 patients (14.6%) were lost to follow-up regarding their birth details.

**Table 5:** Parental-fetal outcomes by timing of intervention

	Antepartum (*n* = 34)			Postpartum (*n* = 14)
	1st trimester (*n* = 7)	2nd trimester (*n* = 22)	3rd trimester (*n* = 5)	
Days between intervention and delivery, median (range)^†^	202 (187 to 241)	149 (72 to 189)	41 (6 to 81)	27 (1 to 93)
Parental outcomes				
Induction for biliary disease				4 (28.6%)
Intrapartum hemorrhage	0	1 (6.3%)	0	1 (7.7%)
Postpartum hemorrhage	1 (14.3%)	0	0	0
Intrahepatic cholestasis of pregnancy	0	0	1 (25%)	0
Preeclampsia	2 (28.6%)	2 (11.8%)	1 (25%)	3 (23.1%)
Eclampsia	0	0	1 (25%)	0
Pregnancy loss	1 (14.3%)	0	0	0
Preterm delivery	0	1 (5.6%)	2 (50%)	3 (21.4%)
Cesarean section for fetal distress	0	3 (17.6%)	0	2 (15.4%)
Fetal outcomes				
Birthweight (g), median (range)	3320 (446 to 3960)			3137.5 (2070 to 3900)
	3490 (3285 to 3620)	3330 (446 to 3960)	2736 (2268 to 3570)	
Small for gestational age	0	2 (12.5%)	0	1 (8.3%)
Large for gestational age	0	0	0	1 (8.3%)
Apgar (1 min), median (range)	8 (1 to 9)			8 (4 to 9)
	9 (7 to 9)	8 (1 to 9)	8 (8 to 9)	
Apgar (5 min), median (range)	9 (6 to 9)			9 (8 to 9)
	9 (9 to 9)	9 (6 to 9)	9 (9 to 9)	
Fetal growth restriction	0	2 (11.8%)	0	1 (8.3%)
NICU admission	0	2 (12.5%)	1 (25%)	5 (41.7%)

^†^Days between intervention and delivery calculated after excluding pregnancy losses at less than 20 weeks gestation

## Discussion

To our knowledge, this is one of few studies to leverage a multi-hospital health system electronic medical system to provide an in-depth analysis of complicated biliary disease during pregnancy. Our findings confirm that procedural intervention for complicated biliary disease is safe across all trimesters. We also provide new information on health resource utilization for patients with biliary disease at the later stages of pregnancy.

Studies from the last two decades have demonstrated that laparoscopic cholecystectomy (LC) is safe to perform in all trimesters [10, 11, 24]. The safety and efficacy of endoscopic retrograde cholangiopancreatography (ERCP) in pregnancy has also been previously established [25, 26]. As such, current guidelines for the management of complicated biliary disease during pregnancy recommend treatment with LC or ERCP across all trimesters [12, 13]. Our study cohort included patients who underwent LC for acute cholecystitis, and a combination of ERCP and/or cholecystectomy for choledocholithiasis, cholangitis, and gallstone pancreatitis. Our findings confirm equivalent surgical and parental-fetal outcomes across all trimesters of antepartum intervention, with any variability likely attributable to low sample sizes within each trimester cohort.

For patients diagnosed with complicated biliary disease in pregnancy, non-operative management (NOM) has been associated with higher rates of preterm delivery, labor complications, and pregnancy loss, and increased rates of fetal complications due to operative delay [8, 9, 27]. In our study, parental-fetal outcomes such as the incidence of preeclampsia and pregnancy loss were largely similar across groups. One notable exception in our data was the proportion of NICU admissions, which was higher in the postpartum intervention cohort compared to the entire antepartum cohort. There also seemed to be a higher proportion of preterm deliveries in the postpartum intervention cohort. Although these differences may be attributable to low sample sizes, potential risks to the fetus cannot be ignored when pursuing NOM, and patients should be counseled accordingly. Our study provides additional granularity to the existing literature in demonstrating that four patients had to be induced after initial NOM of biliary colic because of the severity of their complicated biliary disease on readmission. In these cases, early operative management could have alleviated the role that biliary disease played in peripartum decision-making.

Timely procedural intervention of biliary disease in pregnancy is necessary. Biliary colic managed non-operatively can eventually progress to complicated biliary disease, which in turn is associated with an increased risk of both surgical and pregnancy-related complications [6, 7]. In one retrospective study, 27% of recurrences after diagnosis of biliary colic in pregnancy involved complications such as acute cholecystitis, cholangitis, or pancreatitis [28]. Our study mirrors those findings as 29% of our cohort required postpartum intervention due to progression of their biliary disease from an uncomplicated to complicated state within 90 days of delivery.

Few studies have examined hospital resource utilization, and existing data on the topic is conflicting. One retrospective cohort study by Othman et al. found that NOM is associated with more frequent visits to the emergency department [21]. Some analyses of large inpatient databases have suggested that cholecystectomy during pregnancy is linked to increased length of stay and higher readmission rates, while others have called for cholecystectomy at index antepartum admission to reduce cumulative hospital costs [8, 29, 30]. Our study expands on the current literature by including outpatient, emergency department, and obstetric triage visits, providing a novel level of granularity. Our findings highlight that resource utilization was equivalent at all levels of care when comparing antepartum and postpartum intervention. However, among patients receiving antepartum intervention, 3rd trimester patients required the highest level of healthcare resource utilization, as evidenced by longer total antepartum length of stay and post-surgical length of stay. These differences could reflect a need for careful fetal monitoring later in pregnancy. Nevertheless, 3rd trimester patients seem to be exposed to increased financial impact compared to patients in earlier trimesters.

There are several limitations to the current study worth noting. The retrospective design precludes us from drawing conclusions about causality, and the small sample size certainly constrained our ability to detect statistically significant differences in our results. Additionally, it is worth mentioning that 14.6% (*n* = 7) of patients in our study cohort were lost to follow-up regarding their birth outcomes. Thus, the actual incidence of adverse birth outcomes could have been underestimated in this study. To gain a more comprehensive understanding of the effects of operative treatment, improved follow-up and detailed documentation of birth outcomes are necessary.

In conclusion, our study provides valuable insight into contemporary management of complicated biliary disease during pregnancy within a US tertiary healthcare system. We confirm what is known about the safety of intervention across trimesters and highlight the importance of pursuing procedural management on index admission. We present novel data showing increased healthcare utilization among 3rd trimester patients, and also demonstrate a trend toward recurrence of complicated disease postpartum when intervention is deferred in the 3rd trimester. We believe that next steps should focus on the standardized evaluation of and targeted follow-up strategies for pregnant patients presenting with symptomatic biliary disease. Future research could also involve detailed cost-analysis to better quantify levels of healthcare utilization, as well as multi-institutional collaboration to boost sample sizes and thereby inform clinical guidelines.
